# Joys or Sorrows of Parenting During the COVID-19 Lockdown: A Scoping Review

**DOI:** 10.3389/phrs.2022.1605263

**Published:** 2023-01-04

**Authors:** Marta Fadda, Matilde Melotto, Maria Caiata-Zufferey, Milo Alan Puhan, Anja Frei, Emiliano Albanese, Anne-Linda Camerini

**Affiliations:** ^1^ Institute of Public Health, Università della Svizzera italiana (USI), Lugano, Switzerland; ^2^ Department of Business Economics, Health and Social Care, University of Applied Sciences and Arts of Southern Switzerland (SUPSI), Manno, Switzerland; ^3^ Epidemiology, Biostatistics and Prevention Institute, University of Zurich, Zürich, Switzerland

**Keywords:** COVID-19, pandemic, review, lockdown, parenting

## Abstract

**Objectives:** The aim of this scoping review was to map out the existing evidence of the impact of the COVID-19 lockdown on parents of children and adolescents. We sought to: 1) identify parenting domains that were particularly affected by lockdown measures, 2) describe the challenges and opportunities of lockdown measures in these domains, and 3) define protective and exacerbating factors modulating the effect of lockdown measures on parents.

**Methods:** We identified five main domains investigated in the context of parenting during the early COVID-19 lockdown derived from 84 studies: health and wellbeing, parental role, couple functioning, family and social relationships, and paid and unpaid work. For each domain, we listed challenges and opportunities, as well as discriminant factors.

**Results:** The lockdown impacted all five different but interconnected domains, introduced new roles in parents’ lives, and particularly affected women and vulnerable populations.

**Conclusion:** This scoping review highlights the importance of approaching public health policymaking from a social justice perspective. Such an approach argues for social and public health policies to promote health accounting for its social, economic, political, and commercial determinants.

## Introduction

The management of the early phases of the COVID-19 pandemic has involved aggressive mitigation measures, including stay-at-home restrictions and strict physical distancing, school and workplace closures, a temporary, complete lockdown of businesses, and cancellation of public events, all geared towards reducing the spread and impact of infections [1–4]. The World Health Organization recommends that countries update their pandemic preparedness plans based on lessons learnt from previous outbreaks and latest evidence available on the effectiveness of containment and mitigation measures [5]. However, while evidence is growing on their effectiveness, less is known on the impact of such measures [6–11]. Most studies to date have focused on the impact of mitigation measures, particularly lockdown measures, on older adults [12–16] and children and adolescents [17–24].

Other populations were affected during the lockdown, too, but received less attention in the scientific and public debate. Notably, abrupt school/childcare closures and employment instability created unprecedented conditions for parents, who had to manage their children and adolescents at home 24 h a day while, at the same time, moving to smart-working. Previous literature reviews and meta-analyses on the impact of the lockdown on parents focus mainly on single issues, such as its impact on mental health or parental employment [8, 25–27], or specific populations, such as parents with children or adolescents with special needs [28–30]. The diversity of scope, methods and settings makes this corpus of evidence hard to assemble to provide a complete picture of the impact of the lockdown on families. However, pandemic preparedness plans would benefit from a broad scoping review aimed at mapping the diverse scientific evidence on the outcomes of strict lockdown measures on several key domains in parents’ lives, the structural vulnerabilities worsening outcomes among certain groups, the protective factors leading to better outcomes, and the possible opportunities, if any, that public health policies to contain the spread of COVID-19 might have introduced.

### Objectives

The aim of this scoping review was to map out the existing evidence of the impact of the COVID-19 lockdown on parents of children and adolescents at multiple levels [31]. More specifically, we aimed to:

1) identify the parenting domains that were particularly affected by lockdown measures,
2) describe the challenges and opportunities of lockdown measures in these domains, and
3) define protective and exacerbating factors modulating the effect of lockdown measures on parents.

## Methods

We conducted a scoping review using Arksey and O’Malley’s proposed framework [32]. This framework appropriately captures broad topics that may require a wide range of study designs. Design diversity is generally not suitable for systematic reviews or meta-analyses [31]. We searched for studies that reported on the impact of the lockdown on parents of children and adolescents living at home. Throughout this review, we use the term “parent” to refer to both parental and non-parental guardians of children and adolescents (i.e., caregivers).

### Information Sources

Between 1 and 3 February 2021, we searched the following databases for peer-reviewed studies: Communication & Mass Media Complete, Psychology and Behavioral Sciences Collection, PsycINFO, CINAHL (through EBSCOhost); ERIC: Educational Resource Informatic Center, Medline, ProQuest Sociology (through PROQUEST); PubMed; Web of Science; and Scopus.

ALC, MF and MM identified relevant bibliographic databases, and developed, pilot-tested, refined, and adapted the keywords and search strategy in the various databases. The selected databases cover a wide range of discipline including human health, public health, health systems, sociology, communication, and psychology.

### Search Strategy

The search strategy involved formulating keywords and Medical Subject Headings (MeSH) starting from the PICO elements of our research question and focusing on three main themes of this study: 1) parenting (e.g., legal guardians, parents), 2) COVID-19 pandemic (e.g., outbreak), and 3) lockdown (e.g., confinement) (see Supplementary Table S1). We intentionally did not include keywords related to the outcome component of the research question to comprehensively capture a wide range of protective and exacerbating factors as well as psychological, social, contextual, and behavioral outcomes. We restricted the database searches to peer-reviewed articles in English published between January 2020 and January 2021. We exported all retrieved records in the Zotero reference software to remove duplicates.

### Eligibility Criteria of Included Studies

We included published, English-language, peer-reviewed studies reporting findings from quantitative, qualitative, or mixed-methods studies or original analysis of evidence. We assessed the relevance of the retrieved studies to ensure that they related to caregivers of children and adolescents, who spent the first 6 months of the pandemic in the same household. Because our focus was on evidence of the occurrence and nature of the impact of lockdown measures, we excluded randomized-controlled trials, case studies, (auto) ethnographies, (auto) biographies, unpublished study reports, preprints, papers published in non-peer-reviewed journals, commentaries and opinion pieces, reviews, studies targeting children/adolescents only or pregnant women, studies targeting children/adolescents and parents but with no focus on parental outcomes, studies conducted in hospital settings, and non-English-language articles.

### Study Selection, Categorization, and Data Extraction

We employed an iterative approach to select, categorize, and extract data from the retrieved studies. At first, two coders (MF and MM) reviewed all titles and abstracts derived from the search. Discrepancies were resolved through discussion led by a third, independent coder (ALC). To assess the level of agreement between the first two coders, we computed an interrater reliability score using Cohen’s kappa statistics. The full text of the included articles was then screened for eligibility. From each article included in the review, we extracted the first author’s name, year and month of publication, country of origin, sample size, study design and data collection methodology. An inductive, analytic approach was used to identify the investigated domains by deriving themes from the articles and related sub-themes (Objective 1), while a deductive approach was employed to categorize challenges and opportunities of the lockdown in the extracted domains (Objective 2), and respective protective and exacerbating factors (Objective 3).

## Results

**FIGURE 1:**
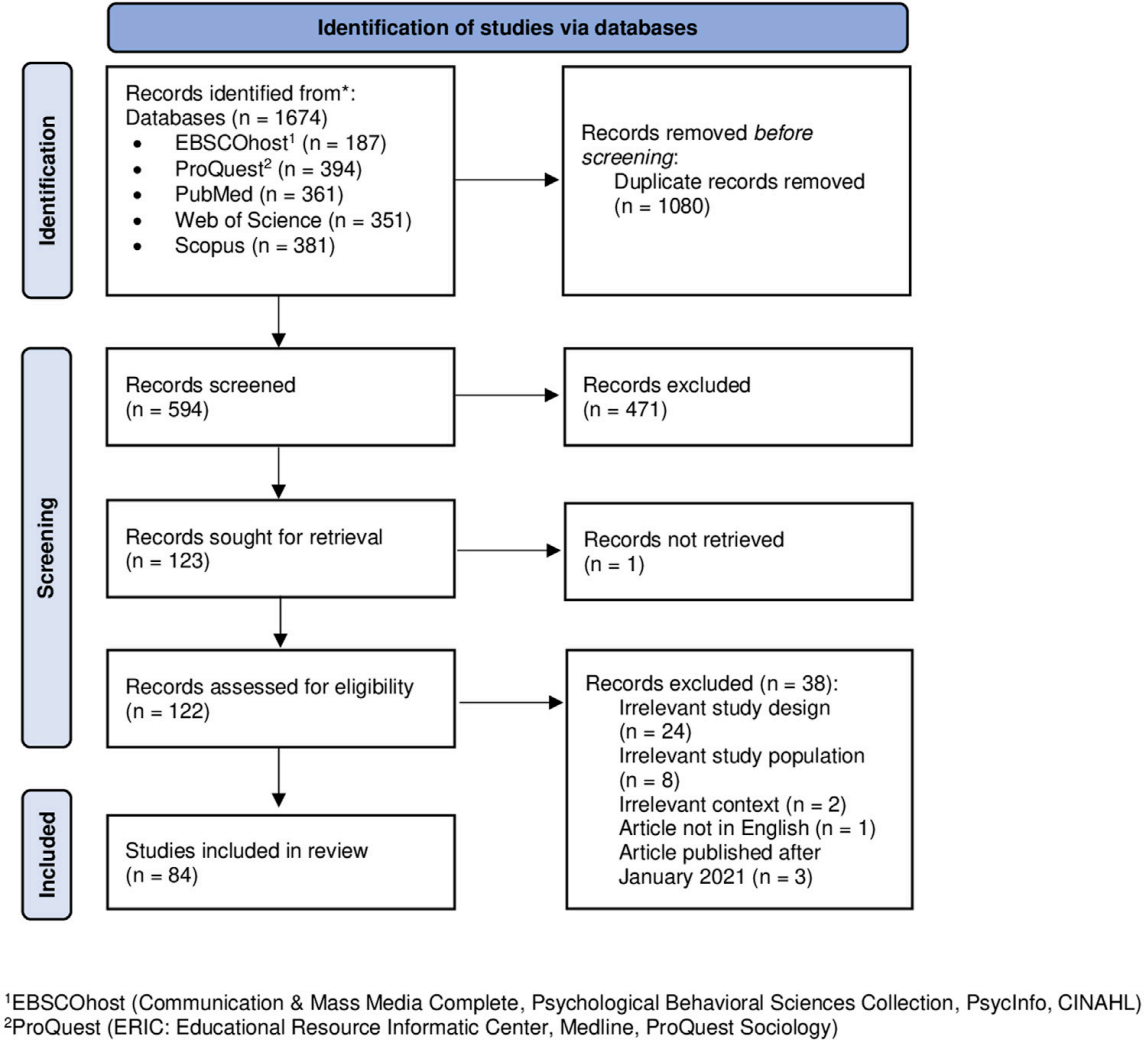
Preferred Reporting Items for Systematic Reviews and Meta-Analyses (PRISMA) flow diagram (Lugano, Switzerland. 2022).

The database search returned 1,674 records. Of these, 1,080 were duplicates and thus discarded. We screened the titles and abstracts of the remaining 594 records, of which 471 did not meet the inclusion criteria and were excluded. With a Cohen’s kappa of 0.67 (87% agreement), intercoder reliability for the two coders (MF and MM) was substantial [33]. We obtained the full texts and screened the eligibility of the remaining 122 publications. After the exclusion of 48 of them, we retained 84 publications for data abstraction. The updated Preferred Reporting Items for Systematic Reviews and Meta-Analyses (PRISMA) flow diagram [34] in Figure 1 lays out these procedures in more detail.

### Study Characteristics

Of the 84 included articles, 17 (20%) were published between July and September 2020, half (43/84, 51%) between October and December 2020, and 20% (17/84) in January 2021. Studies were conducted in 29 countries. Italy was the most represented (21/84, 25%), followed by the United Kingdom (10/84, 12%), Germany (8/84, 9.5%), United States (6/84, 7%), and Canada (5/84, 6%). Sixty-six studies were quantitative (79%), 12 qualitative (14%), and six used mixed methods (7%). Most quantitative and mixed method studies (60/72) applied a cross-sectional design, while eleven were longitudinal, and one included both a cross-sectional and a longitudinal component. The sample size of quantitative studies varied considerably with a range from 19 to 5,500 participants. Eight qualitative studies used (semi-structured) interviews to obtain data from 5 to 77 parents. The remaining four qualitative studies analyzed open-ended questions from survey responses of 68–2,130 parents. Of the mixed-methods studies, four combined a cross-sectional survey with a qualitative analysis of open-ended questions, one a cross-sectional survey with interviews, and one a longitudinal survey with semi-structured interviews. The percentage of female participants included in the studies ranged from 36.5% to 100%, with 18 studies (21%) including only female participants. Table 1 summarizes the study characteristics (for more details see Supplementary Table S2).

**TABLE 1:** Summary of study characteristics (Lugano, Switzerland. 2022).

Study characteristic	Categories	N (%)
Publication period (q/y)	Q1/2020	—
	Q2/2020	3 (4%)
	Q3/2020	17 (20%)
	Q4/2020	43 (51%)
	Q1/2021	17 (20%)
Continent of data collection	Europe	59 (70%)
	Middle East	3 (4%)
	Asia	12 (14%)
	Northern America	11 (13%)
	Central/South America	2 (2%)
	Africa	1 (1%)
	Oceania	4 (5%)
Study design	Quantitative	66 (79%)
	Qualitative	12 (14%)
	Mixed methods	6 (7%)
Sample size	Up to 100	18 (22%)
	101 to 1,000	40 (48%)
	1,001 or more	25 (30%)
Study population	Parents of children without health conditions or non-specified	69 (82%)
	Parents of children with health conditions	10 (12%)
	Both above categories	5 (6%)
Domains	Health and wellbeing	66 (79%)
	Parenting	43 (51%)
	Couple functioning	10 (12%)
	Family and social relationships	20 (24%)
	Paid and unpaid work	30 (36%)

### Extracted Domains

**FIGURE 2:**
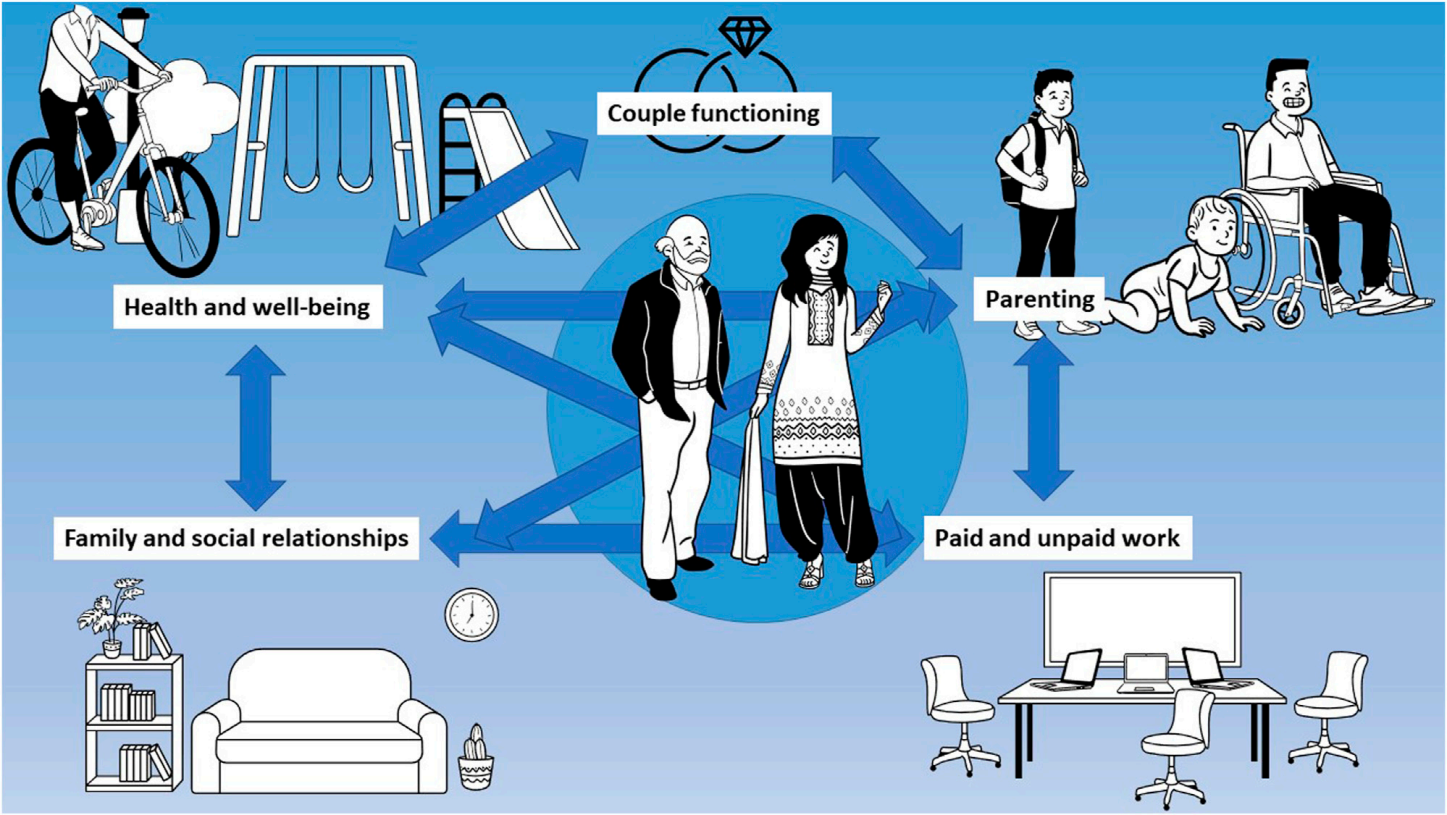
Key domains in parenting affected by the early COVID-19 lockdown (Lugano, Switzerland. 2022).

We identified five main domains investigated in the context of parenting during the early COVID-19 lockdown: health and wellbeing, parenting, couple functioning, family and social relationships, and paid and unpaid work. We further divided each domain into subdomains, where applicable, to list challenges and opportunities, as well as any sources of disparities (see also Figure 2).

#### Health and Wellbeing

##### Mental Health

Studies consistently reported that mental health worsened among parents during the lockdown. Parents with one or two children reported lower levels of mental health compared to people without children [35]. Depression and anxiety increased among mothers compared to the pre-lockdown period [36], and mothers reported higher scores during the lockdown when compared to fathers [37]. Unexpected anxieties, depression, and loneliness were linked to disruption of social support and routine [38] and caring for children with Special Educational Needs and/or Disabilities (SEND), who reported receiving significantly less social support than other parents [39–42]. Greater parenting-related exhaustion and stress, and deteriorated mental health in general, were more common among mothers [43–48], parents under age 35, parents with a pre-existing mental or physical health condition or a disability, receiving psychological treatment [43, 47, 49, 50], parents reporting unemployment, financial stress, change in employment, or a job role in business [43, 47, 49–52], parents reporting fewer perceived social connections or loss of socialization [46, 53], and divorced/separated parents [46, 49, 54]. Parents of children with disabilities experiencing financial difficulties reported sleepless nights, loss of appetite, and anger [55]. Mental health issues were sometimes found to be related to a worsening of existing mental health difficulties, in other cases the lockdown was associated with new mental health problems and symptoms [56]. Moreover, a phenomenon of “emotional contagion” was found among family members (particularly in families with children with disabilities or particular conditions), where a depressed or anxious individual influenced the mental health of the other family members [56]. Poor mental health was significantly more prevalent among parents of younger children (≤4 years) [43, 45, 50], parents with a large number of children [46], parents of children with intellectual disability [39, 40], developmental disorders [51], psychological, physical, or genetic diseases [45], with moderate or high emotional distress or mental illness [47], or behavioral and emotional difficulties [54]. By contrast, parents working in the healthcare sector [44] and fathers had higher scores in psychological wellbeing [57], although one study showed that the former were more likely to report work-related distress [47]. Parents with a supportive employer identified this as a factor that helped managing their stress related to the lockdown [43].

Studies exclusively looking at mothers found that higher age, lower income, poorer physical health, experiencing work suspension and lower perceived maternal support, and being a single mother were associated with more stress [58, 59]. Conversely, protective factors lowering the risk for mental health symptoms included being married, living in rural areas, and having larger families and support [58, 60, 61]. However, high education and high family income were found to relate to high levels of mental health symptoms among mothers living in China [60]. Mothers tried to strengthen their emotional resilience by adopting a variety of psychological strategies, e.g., being optimistic and suppressing negative thoughts [62]. Household income and house size were strongly related to mental health outcomes for parents of children with intellectual disabilities, which tended to improve with better financial means and social support [39].

##### Lifestyle Behaviors

Parents reported some leisure activities and events were missing in the new daily family life during lockdown [56]. The risk for adverse changes in some dietary behaviors was significantly higher among parents experiencing food insecurity and among single parent families with children compared to single persons and couples without children [63]. Feeding on schedule decreased on average, but a smaller decrease was observed in more educated parents [64]. Less educated parents also tended to serve larger portions and set less restrictions for unhealthy food [64]. Few parents of children affected by mental health conditions reported increased alcohol/drug use during the lockdown compared to the period before [41]. Parents with low educational background were more likely to report a decrease in their general life satisfaction [65].

On the other hand, some parents saw the lockdown as an opportunity to eat healthy, exercise, and lose weight [66–68]. Some enjoyed a slower pace of life and were able to sleep more, go outside more, meditate and reflect [43, 61, 67, 68]. Parents reported the adoption of a healthier diet and lifestyle [68, 69], greater motivation for buying pleasurable, sustainable, and healthy food [64]. Parents with a higher education level reported preparing more homemade, comforting dishes than before, while parents working from home and parents at home without work had lower motivation to buy convenient food [64]. Among mothers with younger children (<6 years), time for physical activity decreased, while sedentary time increased with children’s age [70, 71].

#### Parenting

##### Pedagogical Role

Parents had to perform multiple, unexpected roles during the lockdown. One of these was the educator role usually performed by schoolteachers. The main pedagogical role of teachers is to promote student learning. The teacher needs to be capable in terms of the content to be taught and different ways of student learning, be able to organize lessons, facilitate the interaction among students and solve challenges in learning. While some parents reported that this role allowed them to get more involved in the life of their child and obtain more insight into their child’s learning [72, 73], other studies reported that it was challenging to take over the pedagogical role of the school [74, 75]. Factors that complicated home-schooling were lack of a sufficient number of devices for each member of the family, lack of time and support from the school, and lack of expertise (parents do not feel able to replace teachers, in particular parents have problems in motivating their children) [75]. Parents with children with special learning needs and low-income parents reported greater difficulties due to lack of differentiated instruction for different needs, lack of support, and inadequate digital tools [40, 73, 74, 76].

##### Therapeutic Role

As most medical consultations for children affected by disabilities or disorders were cancelled or postponed during the lockdown, children required significant parental help. Parents reported performing different types of therapy for their children affected by disability [77], rationing insulin and glucose test strips for their children with type 1 diabetes [78], and facing challenges in accessing health and rehabilitation services and medication. Parents experienced great distress as they noticed their child’s health worsening without having access to appropriate medical care (particularly among parents from rural backgrounds) [28, 55, 76, 79]. Parents with lower education perceived more difficulties meeting their child’s basic care needs than those with a college or university degree [80].

##### Psycho-Social Role

Parents expressed concerns about their children’s emotional, social, and cognitive developments and their children’s growing use of screen media [56, 81]. Few parents reported interpersonal conflict with their child during the lockdown [51]. Mothers reported concerns about lack of direct external support. In particular, they acknowledged that social contacts would have helped them to understand and perform their role in supporting the psychological wellbeing of their children and provide social support to them [81]. Parents’ perceived difficulty in parenting due to social isolation was found to be significantly associated with risk for child maltreatment [82]. Parents of children with attention deficit hyperactivity disorder (ADHD) reported an increase in irritability, shouting at the child, verbal abuse, and punishments [79].

On the other hand, studies consistently reported that the lockdown allowed parents to spend more time with their children [38, 71, 79, 83]. New mothers reported that the lockdown represented an opportunity to have more freedom to enjoy their babies, to learn about them, to bond with babies alone, and to experience an improved breastfeeding journey [81, 84, 85]. The lockdown allowed parents to be more involved in their children’s lives [69]. The pandemic situation helped them improve their listening, relational, and educational abilities, and enhanced dialogue in the family and the quality of time shared with children [43, 51, 86, 87], and it helped them contribute to the child’s psycho-social development [88]. In some cases, new common interests were developed [56], and children helped parents more in the household chores [79]. Families ate together more often during the lockdown [66], and more time was spent cooking together with children particularly in families with more educated parents [64].

Parents who experienced more loneliness or stress also perceived more adverse change in their parenting and interpersonal difficulties with their children [51, 89]. The meal time atmosphere quality improved during the lockdown, but not for those parents who became more stressed at home [64]. Parents of older children and children affected by autism spectrum disorder (ASD) reported experiencing more difficulty ensuring their child’s cognitive, affective, basic, and security needs were met than parents of younger children [42, 80]. Mothers who experienced financial loss were more likely to have higher child abuse risk scores [89]. Parent-child relationships were more difficult for separated families with shared custody, and some children were not able to access parents who lived across state borders [56].

#### Couple Functioning

In general, conjugal functioning was much worse for couples with children in the household during the lockdown than before [44]. The constant presence of partners sometimes created tensions in the couple relationship [81]. Although being confined at home, parents complained of not having enough time *together*, since they were constantly attending to children [56]. Women living with their husbands shared information about suffering from increased forms of emotional, physical, and sexual violence [36]. Couple deterioration was associated with receiving a psychological treatment or diagnosis of a mental or physical health problem [44, 51, 60], and with pre-existing couple relationship difficulties [56].

Conversely, some studies showed that parents reported that their relationship with the spouse improved during the lockdown or it remained stable [44, 51]. Parents having no emancipated children or preschoolers at home, as well as employed and more educated parents saw higher relational improvement during the lockdown [44]. Finally, fathers had higher scores in marital satisfaction than mothers [57].

#### Family and Social Relationships

One of the difficulties introduced by the lockdown was the loss of support for parents, particularly by grandparents [90]. Other negative aspects regarded the impossibility to share new experiences with the extended family and friends network, and the loss of moments considered important by the participants [81, 83]. Parents reported that family coexistence during the lockdown was moderately difficult due to physical proximity [90], particularly for women who were stuck at home with partners with whom they were separating [38, 90]. Increased difficulties were reported by large families living in small houses without gardens, open spaces or in apartments [56]. Fathers’ family satisfaction increased after changing to short-time work during the lockdown, while mother’s satisfaction decreased [91].

On the other hand, parents reported spending more time together as a family and improvements in family cohesion and expressiveness and a significant reduction of family conflict [56, 69, 86]. Connecting with family and friends *via* phone and video chat or with those in their household, maintaining a family routines, and playing indoors and outdoors were identified as strategies that helped them during lockdown [43]. Parents reported experiencing solidarity among parents, i.e., “systemic resilience” conceived as the support coming from the surrounding environment, outside the family system (e.g., other families) [87].

#### Paid and Unpaid Work

##### Income

During the lockdown, only a minority of families remained food secure, while mild, moderate, and severe forms of food insecurity increased [36]. Some families experienced “money worries,” while others faced serious deprivation, e.g., worries for paying rent or mortgages, and having enough money to buy food or other essentials such as household utilities and medications, or worries related to future needs and possibility to afford them [56, 61]. One study found that 10% of parents lost their jobs and had financial difficulties as a consequence of the lockdown [92]. Mothers experienced relatively harsher financial hardship than fathers [48], and more mothers than fathers extended their working hours in the first months of the pandemic [93].

##### Productivity and Job Satisfaction

In terms of perceived work productivity and job satisfaction, studies found that, before the COVID-19 lockdown, women’s perceived work productivity was as high as men’s, while, during lockdown (and particularly during the early period), women reported being less productive than men and less satisfied with their job [91, 94, 95].

##### Distribution of Work

The lockdown made conciliating paid and unpaid work burdensome and beyond the capabilities of many parents because of an increased amount of tasks concerning the household and the management of the family [56, 75, 90], with some having to work late into the night in order to manage their job and home schooling at the same time [56]. Families particularly missed the support of friends and family who used to help with childcare and chores [56]. The presence of the father at home was experienced positively in terms of support but negatively due to the increased confusion and necessary planning [81]. At times, husbands appeared to be unhelpful and indifferent to cleanliness standards, and they were considered an assistance for women [62].

Studies consistently showed the presence of gender disparities in the distribution of paid and unpaid work during the lockdown. While both mothers and fathers reported spending more hours per day on housework and childcare during the lockdown than before, women reported spending more hours than men in unpaid work, perpetuating or aggravating an already unequal pre-pandemic distribution of tasks [35, 48, 86, 93–96]. Mothers had to organize childcare and home-schooling and complete household chores much more often than fathers and, consequently, they had much less time for paid work, for themselves, and to focus on their career, and they felt more often exhausted, nervous, and insecure than fathers [65, 90, 97]. In one study conducted in Italy, increased participation by fathers overtook that of mothers only when mothers continued to go to their usual place of work and their partner did not work because of the pandemic, but even under these circumstances, fathers were increasingly involved in childcare but not in housekeeping [98].

Contrary to mothers, fathers were more likely to report they had been impacted positively by the lockdown since they got to spend more time with their children [97]. Moreover, while women’s housework was not affected by their partners’ working arrangement during the pandemic, men were more likely to spend additional time on chores when their partners were working [98]. Higher educated parents, healthcare workers, single parents, and parents with primary school children had a significantly greater chance to face increased difficulty combining paid work and childcare during the lockdown [47, 56, 62, 96].

However, one study indicated small shifts toward a more equal division of labor in the early lockdown period, with increased participation in housework and childcare by fathers [99]. Gender gaps in paid vs. unpaid work time, and satisfaction with work-family balance and partner’s share narrowed because the relative increase in childcare was higher for fathers and because more fathers also felt less satisfied during lockdown than they had before [100].

## Discussion

The aim of this scoping review was to map out the existing evidence on the challenges and opportunities of the COVID-19 lockdown on parents of children and adolescents around the globe, accounting for the role of pre-pandemic exacerbating and protective factors. We found that 1) the lockdown impacted five different but interconnected domains in the life of parents, 2) it introduced new roles in parents’ lives, and 3) it particularly affected women and vulnerable populations. In the next paragraphs, we provide a contextualization and interpretation of our findings and assess their implications accounting for the study limitations.

The results of this scoping review extend previous evidence to a population which was not considered to be at high-risk of detrimental effects of the lockdown [1]. We provide a comprehensive map of five, interrelated domains where the early lockdown introduced challenges and opportunities, accounting for exacerbating and protecting factors. For example, worse health and wellbeing were associated with worse parenting experiences, worse couple relationship, worse family and social connectedness, and worse outcomes in terms of paid and unpaid work. Regarding the latter, outcomes differed significantly between men and women. Women experienced a greater loss in terms of market income [101], and fewer women than men were expected to regain employment during the COVID-19 [102]. This suggests that, when assessing the impact of the COVID-19 lockdown, it is reductive to focus on only one domain at the time. The impact of the lockdown should be seen in its complexity, including the fact that it may alter not only single domains but also the delicate connections between them. As most of the included studies were cross-sectional, future reviews will benefit from more longitudinal and (quasi-) experimental studies to detect cumulative or lagged effects, their weight, mechanisms, and causality.

Another finding of this review is that the lockdown introduced the need for parents to perform multiple roles that are usually performed by trained professionals (e.g., teachers, healthcare workers) or peers simultaneously and without any support. In an age of hyper-specialization, where most services are delegated to specialized professionals, parents were asked to learn new roles and switch to a more generalist role suddenly and without any external support. This finding reminds of the importance for parents to adopt a holistic approach to parenting that is aware of all the aspects of the child’s growth. These include biological, psychological, social, and educational aspects [103–105]. The COVID-19 pandemic and the lockdown suddenly and drastically changed the conditions in which families lived. This required flexibility to meet diverse needs pertaining to the biological, psychological, social, and behavioral ramifications of children’s development [1].

The third main result of this review is that the lockdown shined a spotlight on several structural problems of individuals and their family, such as socioeconomic inequities, pre-existing physical and mental conditions, and dysfunctional couple relationships. The degree to which this public health measure affected and exposed parents to risks is clearly related to existing socio-economic and socio-spatial inequities. In addition, although the lockdown represented an opportunity to spend more family time, improve family relationships, and improve one’s lifestyle, this was not an opportunity for all. Women, parents with pre-existing mental or physical illness, parents of children affected by mental or physical illness, single parents and parents with financial precariousness and social exclusion were more likely to suffer from the consequences of the lockdown and less likely to enjoy its benefits. Those who were worse-off were even worse-off, while those who were better-off coped better and also enjoyed positive outcomes of the forced confinement. This finding highlights the role of structural inequities in COVID-19 response measures’ consequences [106]. Our finding adds that structural inequities affected parents not only at an individual but also at an inter-personal level. For example, the unbalanced distribution of paid and unpaid work between mothers and fathers was associated to the fact that mothers adopted most new roles demanded by the lockdown on top of the diverse roles they were already performing. This was, in turn, associated with interpersonal (e.g., trouble within the couple or between parents and children) and individual problems (e.g., worsened mental health). Previous evidence on the intra-household allocation of tasks during crises, including epidemics or economic recessions, showed changes in the gendered division of labours [107, 108]. Such changes also involve parenting, showing that crises can bring greater gender equality and narrow the disparity in both paid and unpaid work hours between mothers and fathers [109].

A final main result is that the majority (i.e., approximately 65%) of the studies included in this review refer to high-income countries. This finding shows the trend of research focusing on Western, educated, industrialized, rich, and democratic (WEIRD) populations also in the context of parenting during the COVID-19 pandemic. Future studies should specifically look at low and lower middle income countries, which contribute to more than half of the world population [110], to investigate how the COVID-19 pandemic has impacted the five main domains in the context of parenting identified in this scoping review and whether new other domains of parenting not evident in WEIRD populations have been affected.

### Limitations

Some shortcomings of this scoping review must be noted. First, we included only English-speaking, peer-reviewed articles. Second, we focused on parents/caregivers only. A more comprehensive review could also consider grey literature and the concurrent impact of the lockdown on both parents and children/adolescents to highlight how it altered their relationship. Third, we included only studies published until January 2021. Future research should extend our search and integrate more recently published studies in our map. Moreover, it is important to note that most of the data reviewed in this article were collected early in the pandemic, during the first half of 2020. Since this time, policy responses have changed to promote psychological, social, and economic recovery among different populations. Forth, our analysis did not take into account the different degrees of severity of lockdown measures among countries and assumed homogeneity of interventions. While strict lockdown measures were almost universally implemented during Spring 2020, they were loosened after the first pandemic wave in many European countries but kept in place, for example, in China that followed a “zero-Covid” strategy. We also did not consider pre-existing gender differences at country-level.

## Conclusion

Whether the benefits of the early COVID-19 lockdown in reducing infections were outweighed by their negative impacts on the economy, social structure, education, and mental and physical health of the population is still debated [111]. As evidence mounts on the benefits and costs of restrictive non-pharmaceutical interventions to control the spread of COVID-19, our results suggest considering the impact of the lockdown as not only multi-layered but also as building on pre-existing vulnerabilities and structural inequities. Our results showed that public health policy may negatively impact parents at multiple levels, and affect some parents more than others, when pre-existing structural injustices are not critically examined and addressed. This scoping review highlights the importance of approaching public health policymaking from a social justice perspective. Such an approach argues for social and public health policies to promote health accounting for its social, economic, political, and commercial determinants [112]. Public health should be concerned with promoting policies and practices that are not blind to inequalities related to ethnicity, class, gender, place, and other factors, and with tackling the underlying structures and mechanisms leading to inequitable (health) outcomes [113].
