# Supplementary material for: Joys or Sorrows of Parenting During the COVID-19 Lockdown: A Scoping Review
Source: Public Health Rev. 2023 Jan 4;43:1605263. doi: 10.3389/phrs.2022.1605263 (PMC9845277; doi:10.3389/phrs.2022.1605263)
Supplement: Supplementary file 1 [file DataSheet1.docx]

**Supplement**

Table 1. Search string

| **Key concepts** | **Search terms^a^** |
| --- | --- |
| Parenting | parent* OR caregiver* OR carer* OR mother* OR father* |
|  | AND |
| Covid-19 pandemic | covid* OR pandemic* OR coronavirus* OR outbreak* OR epidemic* |
|  | AND |
| Lockdown | lock* OR isolat* OR confin* |
|  | NOT |
| Pregnancy/hospitalization | pregnan* OR hospital* |

Note: ^a^Search conducted on title and abstract

Table 2. Overview of included studies (n=84, in alphabetical order)

| **No.** | **First author** | **Month/ Year of publication** | **Country/ies** | **Study design** | **Study method** | **Population and sample size** | **Female participants (%)** | **Age** | **Main domain(s)** |
| --- | --- | --- | --- | --- | --- | --- | --- | --- | --- |
| 1 | Amorim | 09/2020 | Portugal | Quantitative (CS) | Survey | 43 parents of children diagnosed with Autism Spectrum Disorder and 56 parents of normally developed children | N/A | N/A | Health and wellbeing |
| 2 | Balenzano | 2020 (month unknown) | Italy | Quantitative (CS) | Survey | 104 parents with at least one child aged 2 -14 years | 80.8% | Range = 25-59 years (mothers: M = 40.14; SD = 5.81 ; fathers: M = 42.5; SD = 7.5) | Health and wellbeing ; Parenting ; Family and social relationships ; Paid and unpaid work |
| 3 | Benassi | 12/2020 | Italy | Quantitative (CS) | Survey | 1030 women, of whom 516 with children | 100% | M = 38.38; SD = 4.60; Range = 25-50 | Health and wellbeing |
| 4 | Bentenuto | 12/2020 | Italy | Quantitative (CS) | Survey | 82 parents of children with neurodevelopmental disabilities and 82 parents of typically developing children | 89.5% | M = 41.5; SD = 6.5 | Health and wellbeing |
| 5 | Bérubé | 10/2020 | Canada | Quantitative (CS) | Survey | 414 parents of children aged 0-18 years | 85.7% | M = 40.2; Range = 18-71 years | Health and wellbeing ; Parenting |
| 6 | Bıkmazer | 2020 (month unknown) | Turkey | Quantitative (CS) | Survey | 3278 parents of children aged 6-18 with different occupational and psychiatric backgrounds | 75% | M = 40.8, SD = 6.88 | Health and wellbeing ; Parenting |
| 7 | Borah Hazarika | 12/2020 | India | Quantitative (CS) and qualitative | Survey and interviews | Parents belonging to 20 families of children aged 2.5-10 | N/A | Range = 30-45 | Health and wellbeing ; Parenting ; Couple functioning ; Paid and unpaid work |
| 8 | Brom | 07/2020 | Czech Republic | Quantitative (CS) | Survey | 9810 carers of children aged 6-15 | 84% | N/A | Parenting |
| 9 | Bubb | 09/2020 | Norway | Quantitative (CS) | Survey | 779 parents of children aged 6-16 | N/A | N/A | Parenting |
| 10 | Buecker | 11/2020 | Germany | Quantitative (L) | Survey | 4,844 adults including parents | N/A | N/A | Health and wellbeing |
| 11 | Cacioppo | 08/2020 | France | Quantitative (CS) | Survey | 1000 parents of children with physical disabilities aged  0-18 years | 88% | N/A | Parenting |
| 12 | Cahapay | 06/2020 | Philippines | Qualitative | Interview | 5 mothers of at least one child diagnosed with ASD | 100% | M = 43.8 | Parenting |
| 13 | Cellini | 01/2021 | Italy | Quantitative (CS) | Survey | 299 mothers of at least one child aged 6-10 | 100% | M = 40.2; SD = 4.79; Range = 22–54 | Health and wellbeing ; Paid and unpaid work |
| 14 | Chartier | 12/2020 | Belgium | Quantitative (CS) | Survey | 287 parents | 80% | M = 42.38; SD = 7.69 | Health and wellbeing |
| 15 | Cheng | 01/2021 | UK | Quantitative (L) | Survey | 6795 working parents | 57% | M = 43.1; SD = 8.9 | Health and wellbeing ; Parenting ; Paid and unpaid work |
| 16 | Craig | 06/2020 | Australia | Quantitative (CS) | Survey | 1536 dual-earner parents with children under the age of 17 | N/A | N/A | Paid and unpaid work |
| 17 | Crescentini | 10/2020 | Italy | Quantitative (CS) | Survey | 721 parents of at least one typically developing child aged 6-18 | 85.8% | M = 42.80; SD = 5.47 | Health and wellbeing ; Parenting |
| 18 | Cusinato | 11/2020 | Italy | Quantitative (CS) | Survey | 463 parents | 90.50% | M = 43.3; SD = 5.88 | Health and wellbeing |
| 19 | Del Boca | 09/2020 | Italy | Quantitative (L) | Survey | 520 mothers | 100% | M = 43.8; SD=9.21 | Paid and unpaid work |
| 20 | Di Giorgio | 08/2020 | Italy | Quantitative (CS) | Survey | 245 mothers of children between 2-5 | 100% | M = 37.31 years; SD = 4.61; Range = 23–49 | Health and wellbeing |
| 21 | Dib | 10/2020 | UK | Quantitative (CS) | Survey | 1329 mothers of children of 12 months or younger | 100% | M = 31.7; SD = 4.7 | Health and wellbeing ; Paid and unpaid work |
| 22 | Evans | 10/2020 | Australia | Qualitative | Survey with open-ended questions | 2130 parents (> 18 years) of children aged 0–18 years | 81% | M = 38.4; SD = 7.1 | Health and wellbeing ; Parenting ; Couple functioning ; Family and social relationships ; Paid and unpaid work |
| 23 | Ezpeleta | 10/2020 | Spain | Quantitative (L) | Survey | 226 parents of adolescents aged 13 years old | 55% | N/A | Health and wellbeing ; Paid and unpaid work |
| 24 | Feng | 09/2020 | USA | Quantitative (CS) | Survey | 286 parents in dual-career families who were working from home during the lockdown | 49.3% | M = 38.35; SD = 9.61 | Paid and unpaid work |
| 25 | Gadermann | 01/2021 | Canada | Quantitative (CS) | Survey | 618 parents with children <18 years old | 52.4% | M = 43.0; SD = 9.0 | Health and wellbeing ; Parenting ; Couple functioning ; Family and social relationships |
| 26 | Grumi | 11/2020 | Italy | Quantitative (CS) | Survey | 84 parents | N/A | N/A | Health and wellbeing |
| 27 | Günther-Bel | 09/2020 | Spain | Quantitative (CS) and qualitative | Survey with closed- and open-ended questions | 193 adults living with children | 77% | Range = 22-77 | Health and wellbeing ; Couple functioning |
| 28 | Guo | 01/2021 | Netherlands, Italy, China | Quantitative (CS) | Survey | 900 Dutch, 641 Italian, and 922 Chinese mothers aged 18 years or older with at least one child between 1 and 10 years old. | 100% | M = 36.74, SD = 5.58 | Health and wellbeing ; Couple functioning ; Family and social relationships |
| 29 | Hamadani | 08/2020 | Bangladesh | Quantitative (L) | Survey | 2424 mothers | 100% | M = 24.1; SD = 4·8 | Health and wellbeing ; Couple functioning |
| 30 | Herbert | 12/2020 | Australia | Quantitative (CS) | Survey | 158 parents of children aged under 6 years | 97% | N/A | Health and wellbeing ; Paid and unpaid work |
| 31 | Hipp | 10/2020 | Germany | Quantitative (L) | Survey | 2265 parents | 76.50% | Range = 25-54 | Health and wellbeing ; Paid and unpaid work |
| 32 | Hristova | 2020 (month unknown) | 11 different countries | Quantitative (CS) | Survey | 19 Bulgarian women with obesity living and working abroad | 100% | Range = 35-65 | Health and wellbeing |
| 33 | Hull | 07/2020 | Australia | Qualitative | Survey with open-ended questions | 336 mothers, 2 fathers, 1 grandmother of brestfed babies | 99% | N/A | Health and wellbeing ; Parenting |
| 34 | Joy | 12/2020 | Canada | Qualitative | Survey with open-ended questions | 68 mothers of a newborn baby aged 0–12 months | 100% | N/A | Health and wellbeing ; Parenting ; Couple functioning ; Family and social relationships |
| 35 | Kumar | 01/2021 | India | Quantitative (CS) | Survey | 85 parents of children under the age of 18 with access to internet and ability to understand English | 36.5% | Fathers: M = 38.1±5.9 years; Mothers: M = 37.9±7.4 years. | Health and wellbeing |
| 36 | Lee(a) | 10/2020 | USA | Quantitative (CS) and qualitative | Survey with closed- and open-ended questions | 405 parents of at least one child aged 0-12 | 69% | M = 34.41; SD = 7.16; Range = 19-56 | Health and wellbeing ; Parenting |
| 37 | Lee(b) | 01/2021 | USA | Quantitative (CS) | Survey | 283 parents of at least one child aged 0-12 | 58.20% | M = 35; SD = 6.89 (21-56 years) | Health and wellbeing ; Parenting ; Family and social relationships |
| 38 | Maertl | 01/2021 | Germany | Quantitative (CS) | Survey | 1034 adults including 138 parents of at least one child under 6 | 51.3% | M = 45.8; SD = 15.7; Range = 18-74 | Health and wellbeing |
| 39 | Mantovani | 01/2021 | Italy | Quantitative (CS) and qualitative | Survey with closed- and open-ended questions | 3443 parents of children aged between 12 months and 5 years and between 6 and 10 years | 93% | N/A | Health and wellbeing ; Parenting |
| 40 | Marchetti | 10/2020 | Italy | Quantitative (CS) | Survey | 1226 parents of at least one child aged 0–13 | 89% | M = 39.13; SD = 6.77 | Health and wellbeing ; Parenting ; Family and social relationships |
| 41 | Mazza | 08/2020 | Italy | Quantitative (CS) | Survey | 833 parents with at least one child aged 3–13 years | 88.4% | M = 40.61; SD = 6.3; Range = 23–67 years | Health and wellbeing |
| 42 | Mazzucchelli | 11/2020 | Italy | Quantitative (CS) | Survey | 885 adult workers, including parents | 73.10% | M= 46.87; SD = 9.77 | Paid and unpaid work |
| 43 | Mbazzi | 12/2020 | Uganda | Qualitative | Interviews | 39 parents, of which 27 had a child with disabilities | 94.9 | N/A | Health and wellbeing ; Parenting ; Family and social relationships ; Paid and unpaid work |
| 44 | McNeilly | 12/2020 | Scotland | Qualitative | Semi-structured interviews | 10 mothers of children in nursery and primary schools | 100% | N/A | Health and wellbeing ; Parenting ; Couple functioning ; Family and social relationships |
| 45 | Mohring | 10/2020 | Germany | Quantitative (L) | Survey | Up to 2639 members of the general population aged 16-75 including parents | N/A | N/A | Health and wellbeing ; Paid and unpaid work |
| 46 | Morelli | 10/2020 | Italy | Quantitative (CS) | Survey | 277 parents of children aged from 6 to 13 | 89.5% | M = 43.36; SD = 4.76; Range = 30 - 58 | Health and wellbeing ; Parenting |
| 47 | Morgul | 09/2020 | UK | Quantitative (CS) | Survey | 927 carers of children aged 5-11 | 96.9% | M = 39.3, SD = 5.5 (21-61 years) | Health and wellbeing ; Family and social relationships |
| 48 | Mousavi | 12/2020 | Iran | Quantitative (CS) | Survey | 213 parents | 65.7% | Mothers: M = 37.43; SD = 7.22); Fathers: M= 41.95; SD = 8.6 | Health and wellbeing |
| 49 | Neece | 10/2020 | USA | Qualitative | Structured interview | 77 parents of preschool‐aged children (3–5 years old) with developmental delay or autism spectrum disorder (ASD) | 71.4% | M = 37.75; SD = 7.27 | Health and wellbeing ; Parenting ; Family and social relationships ; Paid and unpaid work |
| 50 | O’Hagan | 11/2020 | UK | Quantitative (CS) | Survey | 5500 parents of children with special education needs and disabilities | N/A | N/A | Health and wellbeing ; Parenting |
| 51 | Odeh | 08/2020 | Jordan | Quantitative (CS) | Survey | Parents of 235 children with type 1 diabetes | 50.6% | N/A | Parenting |
| 52 | Ohlbrecht | 12/2020 | Germany | Quantitative (CS) | Survey | 2009 members of the general population, of which one third lived in a household with children under 18 | 71% | Persons under 30 and 30 to 40 years of age are most frequently represented in the sample, each by about one third. 23% are 40-55 years old and 15% are over 55 years old | Health and wellbeing |
| 53 | Ollivier | 12/2020 | Canada | Qualitative | Survey with open-ended questions | 68 mothers of a newborn 0-12 months of age | 100% | Range = 25-35 | Health and wellbeing ; Parenting ; Family and social relationships |
| 54 | Paulauskaite | 01/2021 | UK | Quantitative (CS) | Survey | 88 parents of young children aged 30 to 59 months with moderate to severe developmental delays and challenging behavior | 95.50% | 25– 34 (37%); 35– 44 (45%); 45– 54 (15.9%); 55– 64 (1%) | Health and wellbeing ; Parenting ; Family and social relationships ; Paid and unpaid work |
| 55 | Petrocchi | 11/2020 | Italy | Quantitative (CS) | Survey | 144 mothers of children aged 5-10 | 100% | M = 39.3; SD = 5.6; Range = 25–52 | Health and wellbeing |
| 56 | Philippe | 01/2021 | France | Quantitative (CS) | Survey | 498 parents of children aged 3-12 | 71.70% | Range = 25-64 | Health and wellbeing |
| 57 | Pozas | 01/2021 | Germany and Mexico | Qualitative | Semi-structured interviews | 7 parents of children attending primary school | 86% | N/A | Parenting ; Paid and unpaid work |
| 58 | Prikhidko | 12/2020 | USA | Quantitative (CS) | Survey | 155 parents | 92% | M = 37.2; SD = 8.20 | Health and wellbeing |
| 59 | Ramadhana | 06/2020 | Indonesia | Quantitative (CS) | Survey | 372 parents of students | 52.3% | Less than 40 years old (n = 14); 41–45 years old (n = 75); 46–50 years old (n = 89); 51–55 years old (n = 83); more than 56 years old (n = 104) | Health and wellbeing ; Family and social relationships |
| 60 | Rodriguez | 12/2020 | USA | Quantitative (CS and L) | Survey | 405 parents and 106 mothers of children aged 5-6.5 | 69% and 100% | Participants of the cross-sectional study: M=34; SD=7.17 | Health and wellbeing ; Parenting ; Paid and unpaid work |
| 61 | Sá, de | 11/2020 | Brazil | Quantitative (CS) | Survey | 1352 parents of 816 children aged 0-12 years | N/A | N/A | Health and wellbeing ; Family and social relationships |
| 62 | Sahithya | 11/2020 | India | Quantitative (CS) | Survey | 196 parents of children under 15 | 96% | Mothers: M = 35.60; SD = 7.01; Fathers: M = 40.30, SD = 5.1 | Health and wellbeing ; Parenting ; Couple functioning ; Paid and unpaid work |
| 63 | Santini | 09/2020 | Italy | Quantitative (CS) | Survey | 88 parents of children on Palliative Paediatric Care | 97.70% | N/A | Family and social relationships |
| 64 | Shafer | 2020 (month unknown) | Canada | Quantitative (CS) | Survey | 1245 parents | 50.30% | M = 41.68; SD = 8.81 | Paid and unpaid work |
| 65 | Shah | 01/2021 | N/A | Quantitative (CS) | Survey | 48 parents of children with ADHD | 45.80% | N/A | Parenting |
| 66 | Snuggs | 11/2020 | UK | Quantitative (CS) and qualitative | Survey with closed- and open-ended questions | 86 adults living with children | N/A | N/A | Health and wellbeing ; Paid and unpaid work |
| 67 | Snyder | 01/2021 | USA | Qualitative | Interviews | 29 brestfeeding mothers | 100% | N/A | Health and wellbeing ; Parenting ; Family and social relationships |
| 68 | Spinelli(a) | 07/2020 | Italy | Quantitative (CS) | Survey | 854 parents of children aged between 2-14 | 93.3% | Mothers: M = 38.96; SD = 6.02; Fathers: M = 41.9, SD = 6.75 | Health and wellbeing ; Parenting |
| 69 | Spinelli(b) | 09/2020 | Italy | Quantitative (L) | Survey | 810 parents of children aged 2-14 | 93% | M = 39.09; SD = 5.98 | Health and wellbeing ; Parenting ; Paid and unpaid work |
| 70 | Spinola | 11/2020 | Italy | Quantitative (CS) | Survey | 243 mothers (> 18) of children between 0-1 year | 100% | M = 34; SD = 4.27; Range = 21–47 | Health and wellbeing |
| 71 | Stallard | 01/2021 | Portugal and UK | Quantitative (CS) | Survey | 385 carers of children aged 6-16 | 88.6% | Range = 40-49 | Health and wellbeing ; Parenting ; Family and social relationships ; Paid and unpaid work |
| 72 | Tchimtchoua Tamo | 12/2020 | China | Quantitative (CS) | Survey | 274 mothers | 100% | Range = 24-43 | Health and wellbeing ; Parenting |
| 73 | Thorell | 01/2021 | UK, Sweden, Spain, Belgium, the Netherlands, Germany and Italy | Quantitative (CS) | Survey | 2002 parents with a child aged 5-19 with a mental health condition and 4718 without | N/A | N/A | Health and wellbeing ; Parenting ; Paid and unpaid work |
| 74 | Tierolf | 11/2020 | Netherlands | Quantitative (L) and qualitative | Survey and semi-structured interviews | 246 vulnerable families with at least a child aged 3-18 years | Between 62.6% and 66.3% | Range = 18-55 | Health and wellbeing ; Parenting ; Couple functioning ; Family and social relationships ; Paid and unpaid work |
| 75 | Tokatly Latzer | 01/2021 | Israel | Qualitative | Semi-structured interviews | 31 parents of children diagnosed with ASD during the last 10 years | 81% | N/A | Parenting |
| 76 | Tso | 11/2020 | China | Quantitative (CS) | Survey | 163 parents with pre-schoolers aged 2–5 years and 17,029 parents with school-aged children aged 6–12 years | N/A | Over 60% of fathers and mothers were aged between 35 and 44 years. | Health and wellbeing |
| 77 | Vandevijvere | 12/2020 | Belgium | Quantitative (CS) | Survey | 2160 couples with children and 345 single parents | N/A | N/A | Health and wellbeing ; Paid and unpaid work |
| 78 | Vazquez-Vazquez | 10/2020 | UK | Quantitative (CS) | Survey | 1457 mothers aged ≥18 years with an infant ≤12 months of age | 100% | Mothers who gave birth before LD: M=31.7; SD = 4.6; Mothers who gave birth during LD: M=31.4; SD = 4.9 | Parenting |
| 79 | Venkataraman | 10/2020 | India | Qualitative | Interviews | Upper‐caste urban working women, including mothers | 100% | N/A | Health and wellbeing ; Paid and unpaid work |
| 80 | Wanigasinghe | 12/2020 | Sri Lanka | Quantitative (CS) | Survey | 140 carers of children with epilepsy aged 6 months to 16 years and with at least a diagnosis made 6 months before | 93% | 20-30 (14%), 30-40 (38%), 40-50 (35%), 50-60 (5%), 60+ (3%) | Parenting |
| 81 | Willner | 09/2020 | UK | Quantitative (CS) | Survey | 137 carers (mainly parents) of children with and without intellectual disabilities | 91% | M = 42.1 | Health and wellbeing ; Parenting ; Family and social relationships |
| 82 | Xue | 01/2021 | Switzerland | Quantitative (L) | Survey | 53 parents of term and preterm newborn infants | 81% (During Lockdown); 69% (Before/After Lockdown) | M = 35.8 ± 4.9 (During Lockdown); 35.4 ± 5 (Before/After Lockdown) | Health and wellbeing ; Parenting |
| 83 | Yerkes | 11/2020 | Netherlands | Quantitative (CS) | Survey | 852 parents of at least one child under the age of 18 living at home | N/A | N/A | Parenting ; Paid and unpaid work |
| 84 | Zoch | 10/2020 | Germany | Quantitative (L) | Survey | 785 mothers; 139 mothers and 156 fathers | N/A | N/A | Paid and unpaid work |

Notes: CS = Cross-sectional; L = Longitudinal; NA = Information not available

List of references of the included articles (n=84, in alphabetical order)

1. Amorim R, Catarino S, Miragaia P, Ferreras C, Viana V, Guardiano M. The impact of COVID-19 on children with autism spectrum disorder. *Rev Neurol*. 2020;71(8):285-291. doi:10.33588/rn.7108.2020381
2. Balenzano C, Moro G, Girardi S. Families in the Pandemic Between Challenges and Opportunities: An Empirical Study of Parents with Preschool and School-Age Children. *Ital Sociol Rev*. 2020;10(3S):777-800,777A. doi:10.13136/isr.v10i3S.398
3. Benassi E, Vallone M, Camia M, Scorza M. Women During the Covid-19 Lockdown: More Anxiety Symptoms in Women With Children than Without Children and Role of the Resilience. *Mediterr J Clin Psychol*. 2020;8(3):1-19. doi:10.6092/2282-1619/mjcp-2559
4. Bentenuto A, Mazzoni N, Giannotti M, Venuti P, de Falco S. Psychological impact of Covid-19 pandemic in Italian families of children with neurodevelopmental disorders. *Res Dev Disabil*. 2021;109:N.PAG-N.PAG. doi:10.1016/j.ridd.2020.103840
5. Bérubé A, Clément MÈ, Lafantaisie V, et al. How societal responses to covid-19 could contribute to child neglect. *Child Abuse Negl*. Published online Ottobre 2020. doi:10.1016/j.chiabu.2020.104761
6. Bıkmazer A, Kadak MT, Görmez V, et al. Parental psychological distress associated with COVID-19 outbreak: A large-scale multicenter survey from Turkey. *Int J Soc Psychiatry*. Published online November 5, 2020:20764020970240. doi:10.1177/0020764020970240
7. Borah Hazarika O, Das S. Paid and unpaid work during the covid-19 pandemic: A study of the gendered division of domestic responsibilities during lockdown. *J Gend Stud*. Published online Dicembre 2020. doi:10.1080/09589236.2020.1863202
8. Brom C, Lukavský J, Greger D, Hannemann T, Straková J, Švaříček R. Mandatory Home Education During the COVID-19 Lockdown in the Czech Republic: A Rapid Survey of 1st-9th Graders’ Parents. *Front Educ*. 2020;5. doi:10.3389/feduc.2020.00103
9. Bubb S, Jones MA. Learning from the COVID-19 home-schooling experience: Listening to pupils, parents/carers and teachers. *Improv Sch*. 2020;23(3):209-222. doi:10.1177/1365480220958797
10. Buecker S, Horstmann KT, Krasko J, et al. Changes in daily loneliness for German residents during the first four weeks of the COVID-19 pandemic. *Soc Sci Med 1982*. 2020;265:113541. doi:10.1016/j.socscimed.2020.113541
11. Cacioppo M, Bouvier S, Bailly R, et al. Emerging health challenges for children with physical disabilities and their parents during the COVID-19 pandemic: The ECHO French survey. *Ann Phys Rehabil Med*. Published online Agosto 2020. doi:10.1016/j.rehab.2020.08.001
12. Cahapay MB. How filipino parents home educate their children with autism during covid-19 period. *Int J Dev Disabil*. Published online Giugno 2020. doi:10.1080/20473869.2020.1780554
13. Cellini N, Di Giorgio E, Mioni G, Di Riso D. Sleep and Psychological Difficulties in Italian School-Age Children During COVID-19 Lockdown. *J Pediatr Psychol*. Published online Gennaio 2021. doi:10.1093/jpepsy/jsab003
14. Chartier S, Delhalle M, Baiverlin A, Blavier A. Parental peritraumatic distress and feelings of parental competence in relation to COVID-19 lockdown measures: What is the impact on children’s peritraumatic distress? *Eur J Trauma Dissociation*. 2021;5(2). doi:10.1016/j.ejtd.2020.100191
15. Cheng Z, Mendolia S, Paloyo AR, Savage DA, Tani M. Working parents, financial insecurity, and childcare: mental health in the time of COVID-19 in the UK. *Rev Econ Househ*. Published online Gennaio 2021:1-22. doi:10.1007/s11150-020-09538-3
16. Craig L, Churchill B. Dual‐earner parent couples’ work and care during covid‐19. *Gend Work Organ*. Published online Luglio 2020. doi:10.1111/gwao.12497
17. Crescentini C, Feruglio S, Matiz A, et al. Stuck Outside and Inside: An Exploratory Study on the Effects of the COVID-19 Outbreak on Italian Parents and Children’s Internalizing Symptoms. *Front Psychol*. 2020;11:586074. doi:10.3389/fpsyg.2020.586074
18. Cusinato M, Iannattone S, Spoto A, et al. Stress, Resilience, and Well-Being in Italian Children and Their Parents during the COVID-19 Pandemic. *Int J Environ Res Public Health*. 2020;17(22). doi:10.3390/ijerph17228297
19. Del Boca Daniela, Oggero Noemi, Profeta Paola, Rossi M. Women’s and men’s work, housework and childcare, before and during COVID-19. *Rev Econ Househ*. 2020;18(4):1001-1017. doi:10.1007/s11150-020-09502-1
20. Di Giorgio E, Di Riso D, Mioni G, Cellini N. The interplay between mothers’ and children behavioral and psychological factors during covid-19: An italian study. *Eur Child Adolesc Psychiatry*. Published online Agosto 2020. doi:10.1007/s00787-020-01631-3
21. Dib S, Rougeaux E, Vázquez-Vázquez A, Wells JCK, Fewtrell M. Maternal mental health and coping during the COVID-19 lockdown in the UK: Data from the COVID-19 New Mum Study. *Int J Gynaecol Obstet Off Organ Int Fed Gynaecol Obstet*. 2020;151(3):407-414. doi:10.1002/ijgo.13397
22. Evans S, Mikocka-Walus A, Klas A, et al. From “It Has Stopped Our Lives” to “Spending More Time Together Has Strengthened Bonds”: The Varied Experiences of Australian Families During COVID-19. *Front Psychol*. 2020;11:588667. doi:10.3389/fpsyg.2020.588667
23. Ezpeleta L, Navarro JB, de la Osa N, Trepat E, Penelo E. Life Conditions during COVID-19 Lockdown and Mental Health in Spanish Adolescents. *Int J Environ Res Public Health*. 2020;17(19). doi:10.3390/ijerph17197327
24. Feng Z, Savani K. Covid-19 created a gender gap in perceived work productivity and job satisfaction: implications for dual-career parents working from home. *Gend Manag*. 2020;35(7-8, SI):719-736. doi:10.1108/GM-07-2020-0202
25. Gadermann AC, Thomson KC, Richardson CG, et al. Examining the impacts of the COVID-19 pandemic on family mental health in Canada: findings from a national cross-sectional study. *BMJ Open*. 2021;11(1):1. doi:10.1136/bmjopen-2020-042871
26. Grumi S, Provenzi L, Gardani A, et al. Rehabilitation services lockdown during the COVID-19 emergency: the mental health response of caregivers of children with neurodevelopmental disabilities. *Disabil Rehabil*. 2021;43(1):27-32. doi:10.1080/09638288.2020.1842520
27. Günther‐Bel C, Vilaregut A, Carratala E, Torras‐Garat S, Pérez‐Testor C. A Mixed‐method Study of Individual, Couple, and Parental Functioning During the State‐regulated COVID‐19 Lockdown in Spain. *Fam Process*. 2020;59(3):1060-1079. doi:10.1111/famp.12585
28. Guo J, De Carli P, Lodder P, Bakermans-Kranenburg MJ, Riem MME. Maternal mental health during the covid-19 lockdown in china, italy, and the netherlands: A cross-validation study. *Psychol Med*. Published online Gennaio 2021. doi:10.1017/S0033291720005504
29. Hamadani JD, Hasan MI, Baldi AJ, et al. Immediate impact of stay-at-home orders to control COVID-19 transmission on socioeconomic conditions, food insecurity, mental health, and intimate partner violence in Bangladeshi women and their families: an interrupted time series. *Lancet Glob Health*. 2020;8(11):e1380-e1389. doi:10.1016/S2214-109X(20)30366-1
30. Herbert JS, Mitchell A, Brentnall SJ, Bird AL. Identifying Rewards Over Difficulties Buffers the Impact of Time in COVID-19 Lockdown for Parents in Australia. *Front Psychol*. 2020;11:606507. doi:10.3389/fpsyg.2020.606507
31. Hipp L, Buenning M. Parenthood as a driver of increased gender inequality during COVID-19? Exploratory evidence from Germany. *Eur Soc*. doi:10.1080/14616696.2020.1833229
32. Hristova V. COVID-19 OPPORTUNITIES - BULGARIAN WOMEN: WORK-LIFE BALANCE, OBESITY AND STRESS. *Pedagog-PEDAGOGY*. 2020;92(7, S):134-142.
33. Hull N, Kam RL, Gribble KD. Providing breastfeeding support during the COVID-19 pandemic: Concerns of mothers who contacted the Australian Breastfeeding Association. *Breastfeed Rev*. 2020;28(3):25-35.
34. Joy P, Aston M, Price S, et al. Blessings and Curses: Exploring the Experiences of New Mothers during the COVID-19 Pandemic. *Nurs Rep*. 2020;10(2):207-219. doi:10.3390/nursrep10020023
35. Kumar R, Agarwal N, Gupta A. Knowledge, Awareness and Anxiety towards Coronavirus Pandemic among Indian Parents: A Web-Based Survey. *J Clin Diagn Res*. 2021;15(1). doi:10.7860/JCDR/2021/45824.14420
36. Lee SJ, Ward KP, Chang OD, Downing KM. Parenting activities and the transition to home-based education during the COVID-19 pandemic. *Child Youth Serv Rev*. 2021;122:105585. doi:10.1016/j.childyouth.2020.105585 [Lee(a)]
37. Lee SJ, Ward KP, Lee JY, Rodriguez CM. Parental social isolation and child maltreatment risk during the covid-19 pandemic. *J Fam Violence*. Published online Gennaio 2021. doi:10.1007/s10896-020-00244-3 [Lee(a)]
38. Maertl T, De Bock F, Huebl L, et al. Physical Activity during COVID-19 in German Adults: Analyses in the COVID-19 Snapshot Monitoring Study (COSMO). *Int J Environ Res Public Health*. 2021;18(2). doi:10.3390/ijerph18020507
39. Mantovani S, Bove C, Ferri P, Manzoni P, Cesa Bianchi A, Picca M. Children ‘under lockdown’: voices, experiences, and resources during and after the COVID-19 emergency. Insights from a survey with children and families in the Lombardy region of Italy. *Eur Early Child Educ Res J*. Published online 2021. doi:10.1080/1350293X.2021.1872673
40. Marchetti D, Fontanesi L, Mazza C, et al. Parenting-Related Exhaustion During the Italian COVID-19 Lockdown. *J Pediatr Psychol*. 2020;45(10):1114-1123. doi:10.1093/jpepsy/jsaa093
41. Mazza C, Ricci E, Marchetti D, et al. How Personality Relates to Distress in Parents during the Covid-19 Lockdown: The Mediating Role of Child’s Emotional and Behavioral Difficulties and the Moderating Effect of Living with Other People. *Int J Environ Res Public Health*. 2020;17(17). doi:10.3390/ijerph17176236
42. Mazzucchelli S, Bosoni ML, Medina L. The Impact of COVID-19 on Family Relationships in Italy: Withdrawal on the Nuclear Family. *Ital Sociol Rev*. 2020;10(3S):687-709,687A. doi:10.13136/isr.v10i3S.394
43. Mbazzi FB, Nalugya R, Kawesa E, et al. The impact of COVID-19 measures on children with disabilities and their families in Uganda. *Disabil Soc*. doi:10.1080/09687599.2020.1867075
44. McNeilly H, Reece KM. ‘Everybody’s Always Here with Me!’ Pandemic Proximity and the Lockdown Family. *Anthropol Action*. 2020;27(3):18-21. doi:10.3167/aia.2020.27030
45. Mohring K, Naumann E, Reifenscheid M, et al. The COVID-19 pandemic and subjective well-being: longitudinal evidence on satisfaction with work and family. *Eur Soc*. doi:10.1080/14616696.2020.1833066
46. Morelli M, Cattelino E, Baiocco R, et al. Parents and Children During the COVID-19 Lockdown: The Influence of Parenting Distress and Parenting Self-Efficacy on Children’s Emotional Well-Being. *Front Psychol*. 2020;11:584645. doi:10.3389/fpsyg.2020.584645
47. Morgul E, Kallitsoglou A, Essau CA. Psychological effects of the COVID-19 lockdown on children and families in the UK. *Rev Psicol Clin CON NINOS Adolesc*. 2020;7(3):42-48. doi:10.21134/rpcna.2020.mon.2049
48. Mousavi SF. Psychological Well-Being, Marital Satisfaction, and Parental Burnout in Iranian Parents: The Effect of Home Quarantine During COVID-19 Outbreaks. *Front Psychol*. 2020;11:553880. doi:10.3389/fpsyg.2020.553880
49. Neece C, McIntyre LL, Fenning R. Examining the impact of COVID-19 in ethnically diverse families with young children with intellectual and developmental disabilities. *J Intellect Disabil Res JIDR*. 2020;64(10):739-749. doi:10.1111/jir.12769
50. O’Hagan B, Kingdom S. Experiences of children with special educational needs and disabilities and their families in the United Kingdom during the coronavirus pandemic. *TIZARD Learn Disabil Rev*. 2020;25(4):229-235. doi:10.1108/TLDR-09-2020-0025
51. Odeh R, Gharaibeh L, Daher A, Kussad S, Alassaf A. Caring for a child with type 1 diabetes during COVID-19 lockdown in a developing country: Challenges and parents’ perspectives on the use of telemedicine. *Diabetes Res Clin Pract*. 2020;168:N.PAG-N.PAG. doi:10.1016/j.diabres.2020.108393
52. Ohlbrecht H, Jellen J. Unequal tensions: the effects of the coronavirus pandemic in light of subjective health and social inequality dimensions in Germany. *Eur Soc*. doi:10.1080/14616696.2020.1852440
53. Ollivier R, Aston DM, Price DS, et al. Mental Health & Parental Concerns during COVID-19: The Experiences of New Mothers Amidst Social Isolation. *Midwifery*. 2021;94:102902. doi:10.1016/j.midw.2020.102902
54. Paulauskaite L, Farris O, Spencer H, Absoud A. My son can’t socially distance or wear a mask: How families of preschool children with severe developmental delays and challenging behavior experienced the covid-19 pandemic. *J Ment Health Res Intellect Disabil*. Published online Gennaio 2021. doi:10.1080/19315864.2021.1874578
55. Petrocchi S, Levante A, Bianco F, Castelli I, Lecciso F. Maternal Distress/Coping and Children’s Adaptive Behaviors During the COVID-19 Lockdown: Mediation Through Children’s Emotional Experience. *Front Public Health*. 2020;8:587833. doi:10.3389/fpubh.2020.587833
56. Philippe K, Chabanet C, Issanchou S, Monnery-Patris S. Child eating behaviors, parental feeding practices and food shopping motivations during the COVID-19 lockdown in France: (How) did they change? *Appetite*. 2021;161:105132. doi:10.1016/j.appet.2021.105132
57. Pozas M, Letzel V, Schneider C. ‘Homeschooling in times of corona’: exploring Mexican and German primary school students’ and parents’ chances and challenges during homeschooling. *Eur J Spec Needs Educ*. Published online 2021. doi:10.1080/08856257.2021.1874152
58. Prikhidko A, Long H, Wheaton MG. The Effect of Concerns About COVID-19 on Anxiety, Stress, Parental Burnout, and Emotion Regulation: The Role of Susceptibility to Digital Emotion Contagion. *Front Public Health*. 2020;8:567250. doi:10.3389/fpubh.2020.567250
59. Ramadhana MR. A dataset for emotional reactions and family resilience during COVID-19 isolation period among Indonesian families. *Data Brief*. 2020;31:105946. doi:10.1016/j.dib.2020.105946
60. Rodriguez CM, Lee SJ, Ward KP, Pu DF. The Perfect Storm: Hidden Risk of Child Maltreatment During the Covid-19 Pandemic. *Child Maltreat*. Published online Dicembre 2020:1077559520982066. doi:10.1177/1077559520982066
61. Sá CDSC de, Pombo A, Luz C, Rodrigues LP, Cordovil R. COVID-19 SOCIAL ISOLATION IN BRAZIL: EFFECTS ON THE PHYSICAL ACTIVITY ROUTINE OF FAMILIES WITH CHILDREN. *Rev Paul Pediatr Orgao Of Soc Pediatr Sao Paulo*. 2020;39:1. doi:10.1590/1984-0462/2021/39/2020159
62. Sahithya BR, Kashyap RS, Roopesh BN. Perceived stress, parental stress, and parenting during covid-19 lockdown: A preliminary study. *J Indian Assoc Child Adolesc Ment Health*. 2020;16(4):44-63.
63. Santini A, Avagnina I, Salamon E, Giacomelli L, Shah A, Benini F. How the COVID‐19 lockdown affected the parents of offspring who needed palliative care in the Veneto region of Italy. *Acta Paediatr*. 2020;109(12):2674-2676. doi:10.1111/apa.15560
64. Shafer K, Scheibling C, Milkie MA. The Division of Domestic Labor before and during the COVID‐19 Pandemic in Canada: Stagnation versus Shifts in Fathers’ Contributions. *Can Rev Sociol*. 2020;57(4):523-549. doi:10.1111/cars.12315
65. Shah R, Raju VV, Sharma A, Grover S. Impact of COVID-19 and Lockdown on Children with ADHD and Their Families-An Online Survey and a Continuity Care Model. *J Neurosci Rural Pract*. 2021;12(1):71-79. doi:10.1055/s-0040-1718645
66. Snuggs S, McGregor S. Food & meal decision making in lockdown: How and who has Covid-19 affected? *Food Qual Prefer*. 2021;89:104145. doi:10.1016/j.foodqual.2020.104145
67. Snyder K, Worlton G. Social Support During COVID-19: Perspectives of Breastfeeding Mothers. *Breastfeed Med*. 2021;16(1):39-45. doi:10.1089/bfm.2020.0200
68. Spinelli M, Lionetti F, Pastore M, Fasolo M. Parents’ Stress and Children’s Psychological Problems in Families Facing the COVID-19 Outbreak in Italy. *Front Psychol*. 2020;11:1713. doi:10.3389/fpsyg.2020.01713 [Spinelli(a)]
69. Spinelli M, Lionetti F, Setti A, Fasolo M. Parenting stress during the covid‐19 outbreak: Socioeconomic and environmental risk factors and implications for children emotion regulation. *Fam Process*. Published online Settembre 2020. doi:10.1111/famp.12601 [Spinelli(b)]
70. Spinola O, Liotti M, Speranza AM, Tambelli R. Effects of COVID-19 Epidemic Lockdown on Postpartum Depressive Symptoms in a Sample of Italian Mothers. *Front Psychiatry*. 2020;11:589916. doi:10.3389/fpsyt.2020.589916
71. Stallard P, Pereira AI, Barros L. Post-traumatic growth during the COVID-19 pandemic in carers of children in Portugal and the UK: cross-sectional online survey. *BJPsych Open*. 2021;7(1):1. doi:10.1192/bjo.2021.1
72. Tchimtchoua Tamo AR. An analysis of mother stress before and during covid-19 pandemic: The case of china. *Health Care Women Int*. Published online November 19, 2020. doi:10.1080/07399332.2020.1841194
73. Thorell LB, Skoglund C, de la Peña AG, et al. Parental experiences of homeschooling during the covid-19 pandemic: Differences between seven european countries and between children with and without mental health conditions. *Eur Child Adolesc Psychiatry*. Published online Gennaio 2021. doi:10.1007/s00787-020-01706-1
74. Tierolf B, Geurts E, Steketee M. Domestic violence in families in the netherlands during the coronavirus crisis: A mixed method study. *Child Abuse Negl*. Published online November 17, 2020. doi:10.1016/j.chiabu.2020.104800
75. Tokatly Latzer I, Leitner Y, Karnieli-Miller O. Core experiences of parents of children with autism during the COVID-19 pandemic lockdown. *Autism Int J Res Pract*. Published online Gennaio 2021:1362361320984317. doi:10.1177/1362361320984317
76. Tso WWY, Wong RS, Tung KTS, et al. Vulnerability and resilience in children during the COVID-19 pandemic. *Eur Child Adolesc Psychiatry*. Published online November 17, 2020:1-16. doi:10.1007/s00787-020-01680-8
77. Vandevijvere S, De Ridder K, Drieskens S, Charafeddine R, Berete F, Demarest S. Food insecurity and its association with changes in nutritional habits among adults during the COVID-19 confinement measures in Belgium. *Public Health Nutr*. Published online Dicembre 2020:1-7. doi:10.1017/S1368980020005005
78. Vazquez-Vazquez A, Dib S, Rougeaux E, Wells JC, Fewtrell MS. The impact of the Covid-19 lockdown on the experiences and feeding practices of new mothers in the UK: Preliminary data from the COVID-19 New Mum Study. *Appetite*. 2021;156. doi:10.1016/j.appet.2020.104985
79. Venkataraman A, Venkataraman A. Lockdown & me …!! Reflections of working women during the lockdown in vadodara, gujarat‐western india. *Gend Work Organ*. Published online November 10, 2020. doi:10.1111/gwao.12572
80. Wanigasinghe J, Jayawickrama A, Hewawitharana G, et al. Experience during COVID-19 lockdown and self-managing strategies among caregivers of children with epilepsy: A study from low middle income country. *Seizure*. 2021;84:112-115. doi:10.1016/j.seizure.2020.12.001
81. Willner P, Rose J, Stenfert Kroese B, et al. Effect of the COVID-19 pandemic on the mental health of carers of people with intellectual disabilities. *J Appl Res Intellect Disabil JARID*. 2020;33(6):1523-1533. doi:10.1111/jar.12811
82. Xue A, Oros V, Marca-Ghaemmaghami PL, et al. New Parents Experienced Lower Parenting Self-Efficacy during the COVID-19 Pandemic Lockdown. *Child Basel Switz*. 2021;8(2). doi:10.3390/children8020079
83. Yerkes MA, André SCH, Besamusca JW, et al. “Intelligent” lockdown, intelligent effects? Results from a survey on gender (in)equality in paid work, the division of childcare and household work, and quality of life among parents in the Netherlands during the Covid-19 lockdown. *PloS One*. 2020;15(11):1. doi:10.1371/journal.pone.0242249
84. Zoch G, Bachmann AC, Vicari B. Who cares when care closes? Care-arrangements and parental working conditions during the COVID-19 pandemic in Germany. *Eur Soc*. doi:10.1080/14616696.2020.1832700
